# Protection Genes in Nucleus Accumbens Shell Affect Vulnerability to Nicotine Self-Administration across Isogenic Strains of Adolescent Rat

**DOI:** 10.1371/journal.pone.0086214

**Published:** 2014-01-22

**Authors:** Hao Chen, Rui Luo, Suzhen Gong, Shannon G. Matta, Burt M. Sharp

**Affiliations:** 1 Department of Pharmacology, University of Tennessee Health Science Center, Memphis, Tennessee, United States of America; 2 Department of Human Genetics, David Geffen School of Medicine, University of California Los Angeles, Los Angeles, California, United States of America; INRA, France

## Abstract

Classical genetic studies show the heritability of cigarette smoking is 0.4–0.6, and that multiple genes confer susceptibility and resistance to smoking. Despite recent advances in identifying genes associated with smoking behaviors, the major source of this heritability and its impact on susceptibility and resistance are largely unknown. Operant self-administration (SA) of intravenous nicotine is an established model for smoking behavior. We recently confirmed that genetic factors exert strong control over nicotine intake in isogenic rat strains. Because the processing of afferent dopaminergic signals by nucleus accumbens shell (AcbS) is critical for acquisition and maintenance of motivated behaviors reinforced by nicotine, we hypothesized that differential basal gene expression in AcbS accounts for much of the strain-to-strain variation in nicotine SA. We therefore sequenced the transcriptome of AcbS samples obtained by laser capture microdissection from 10 isogenic adolescent rat strains and compared all RNA transcript levels with behavior. Weighted gene co-expression network analysis, a systems biology method, found 12 modules (i.e., unique sets of genes that covary across all samples) that correlated (p<0.05) with amount of self-administered nicotine; 9 of 12 correlated negatively, implying a protective role. PCR confirmed selected genes from these modules. Chilibot, a literature mining tool, identified 15 genes within 1 module that were nominally associated with cigarette smoking, thereby providing strong support for the analytical approach. This is the first report demonstrating that nicotine intake by adolescent rodents is associated with the expression of specific genes in AcbS of the mesolimbic system, which controls motivated behaviors. These findings provide new insights into genetic mechanisms that predispose or protect against tobacco addiction.

## Introduction

Genetic factors are pivotal determinants of vulnerability to cigarette smoking. In classical genetic studies, the heritability of various smoking behaviors ranges between 0.4–0.6 [1]–[3]. Recent genome-wide association studies (GWAS) have identified several polymorphisms that are strongly correlated with smoking phenotypes (e.g., number of cigarettes smoked per day). Among these, the cluster of nicotinic acetylcholine receptor subunits (i.e., CHRNA5-CHRNA3-CHRNB4) located in chromosome 15q25 has been detected in many study populations [4]–[7]. However, like many GWAS studies of complex traits, these polymorphisms only explain a small fraction of the variation in smoking behavior [8], [9], implicating a large number of unidentified genetic variations in the vulnerability to smoke tobacco.

Laboratory rodents, especially rat, are frequently used to characterize the behavioral effects of nicotine, the principal psychoactive agent in tobacco smoke [10], [11]. In particular, the operant self-administration (SA) of intravenous (i.v.) nicotine, which achieves pharmacokinetic profiles [12], [13] and plasma levels of nicotine [14] similar to those found in human smokers, has been used to model many nicotine behavioral phenotypes associated with dependence. For example, nicotine SA, in the range of 0.015–0.06 mg/kg/bolus i.v., consistently resulted in inverted U-shaped dose response curves (i.e., operant bar presses vs. nicotine dose; [15]–[20]. Similar to the onset of smoking behavior in adolescent humans, we also have shown that inbred adolescent Lewis rats acquired nicotine SA faster and obtained more nicotine than adults [20]. Many workers have shown that operant behavioral responses for nicotine can be extinguished and nicotine-seeking behavior can be reinstated by nicotine associated cues [21], [22] or stress [23]. Additionally, the role of social learning in smoking initiation has also been modeled [24]. Lastly, Varenicline, a smoking cessation drug, has been shown to reduce rodent nicotine self-administration and drug seeking [25], providing further validation of the model.

Hundreds of inbred rat strains have been generated for genetic analyses [26]. Early studies found drastic differences in nicotine SA profiles among several strains of rats [18], [27], [28]. More recently, we systemically determined i.v. nicotine SA behaviors across a panel of isogenic strains [29]. Confirming previous reports [28], we found Fisher 344 rats were the most resistant, while Lewis rats showed the greatest vulnerability to nicotine SA. Heredity affected nicotine SA (h^2^ = 0.64) to a degree similar to that reported for smoking in humans. These behavioral characteristics indicate the potential strength of this model to identify specific genes that mediate human susceptibility to or protection from smoking cigarettes.

Within the mesolimbic system, the nucleus accumbens shell (AcbS) integrates information from multiple brain regions including dopamine input from the ventral tegmental area (VTA) and glutamate input from cortical regions, amygdala, and hippocampus. This integration is essential for the acquisition and maintenance of motivated behaviors. More specifically, the activation of dopaminergic neurons projecting to AcbS from the ventral tegmental area is critical for the acquisition of nicotine self-administration [30]–[32]. During the acquisition and maintenance of nicotine SA, extracellular levels of dopamine are preferentially increased in AcbS compared to nucleus accumbens core [33], and i.v. nicotine SA is attenuated by ablating accumbal dopamine terminals [34]. Nicotine SA also depends on AcbS receptors for other neurotransmitters such as glutamate and acetylcholine (e.g., mGlu2/3, mGlu5, and nicotinic cholinergic receptors containing alpha6 and beta2 subunits) [35], [36]. The effect of nicotine on AcbS dopamine release depends on rat strain, in that dopamine responses were greater in Lewis than Fisher 344 rats [37]. Gene expression differs in the AcbS of Lewis vs. Fisher 344 rats [28], suggesting that variation in the transcriptome of AcbS neurons may regulate neurochemical responses and behavioral phenotypes.

We therefore hypothesized that differential basal gene expression in AcbS accounts for much of the strain-to-strain variation in nicotine SA among adolescent rats from six inbred strains and four F1 hybrids. To ensure the anatomical accuracy of tissue sampling, we used laser capture microdissection to obtain samples from AcbS. We then used transcriptomic sequencing to identify genome-wide gene expression differences among the ten strains. A systems biology method, Weighted Gene Co-expression Network Analysis (WGCNA), was used to analyze the interaction between AcbS gene networks and nicotine SA behavior. Gene networks that are strongly associated with behavioral variation in the amount of self-administered nicotine were identified. A particular strength of this approach is the identification of genes with differential basal expression that confer either susceptibility or promote resistance to nicotine SA.

## Materials and Methods

### Animals

Rats of six inbred strains (Lewis, Fisher 344, Brown Norway, Spontaneous hypertensive rat, Wistar-Kyoto, Dark Agouti) and four selected F1 hybrids (FL, FS, LS and WL; the two letters represent the initials of the maternal and paternal strains, respectively) were bred on-site. Naïve adolescent male rats were sacrificed on postnatal days 41–42, which coincides with the age nicotine SA started in our previous study [29]. Four rats were used for each strain, and no more than two rats were from the same litter. Brains were removed and immediately frozen and stored in −80°C until further processing. All procedures were conducted in accordance with the *NIH Guidelines Concerning the Care and Use of Laboratory Animals* and were approved by the Animal Care and Use Committee of the University of Tennessee Health Science Center. The nicotine self-administration data analyzed in this study were described in our previous report [29].

### Laser capture microdissection

Brains from naïve adolescent rats were sectioned in a Leica cryostat at 10 µm, and sections were mounted onto uncharged glass slides. Slides were dehydrated by immersion in: 100% methanol (3 min), 95% ethanol (2 min), 100% ethanol (1 min, twice) and finally xylene (5 min, twice), and then air-dried (15 min). Arcturus XT (Life Technologies) was used to capture nucleus accumbens shell, outlined by an overlay of the atlas on the screen. The infrared laser was then used to capture the tissue onto CapSure LCM caps (Life Technologies). From each brain, tissues from five sections, each 50 micron from the next section, were captured bilaterally, starting from Bregma position +1.0. From each rat, 2 bilateral samples were obtained from 5 frozen AcbS sections (10 µm) one used for RNA-seq, another for PCR validation.

### RNA extraction, amplification, and transcriptome sequencing (RNA-seq)

RNA trapped in the CapSure LCM caps was extracted using the PicoPure RNA isolation kit (Life technologies); RNA quality was analyzed using Bioanalyzer (Model 2100, Agilent, Foster City, CA). Total RNA (∼500 pg) extracted from laser-captured neurons was amplified using the Ovation RNA-seq system (NuGEN). As confirmed in our previous study [38], the Ovation amplification procedure eliminated the need to deplete ribosomal RNA. The amplified cDNA from each brain was end repaired using the End-It™ (Epicentre Technologies, Chicago, IL) kit, and ligated to the P1 and P2 adaptors used for SOLiD sequencing. The P2 adapter contains the “barcodes” that allow multiplexing of assays. The standard sequencing protocol, provided by Life technologies was followed thereafter.

### RNA-seq data analysis

RNA-seq data were aligned to the rat reference genome (Baylor 3.4/rn4, Nov. 2004) using SHRiMP2 (Ver 2.2.2) [39] in color space mode with default settings (-I 50,500 -m 20 -i -25 -g -40 -e -10) on a Dell R610 cluster (1U R610s, 12 cores each with 4 GB/core). SHRiMP2 was used because of its high sensitivity, accuracy and reasonable speed. However, SHRiMP2 is not capable of mapping reads that are located on the exon-exon junctions. TopHat (Ver 1.4.0) [40] was used to align the reads to both the reference genome and to all known splice junction sites (based on RefSeq annotation file in GTF format downloaded from UCSC genome browser on April 06 2012). Reads aligned to the splices sites were then selected based on the CIGAR code (N) in the resulting SAM file [41]. The genomic alignments from SHRiMP2 and splice site alignments for each sample were then combined. The Cufflinks program (Ver 0.9.3) [42] was then used to estimate gene expression levels in RPKM. Two samples from FL and FS had now total read counts and were removed from further analysis. RPKM values were processed using SampleNetwork.r [43] for batch effect (i.e. different processing date) removal and normalization.

### Validation of RNA-seq results by real-time PCR

A different set of brain sections were obtained from the same rats used in the RNA-seq experiment. LCM of AcbS was conducted using the method described above to obtain RNA. Real-time quantitative PCR of selected genes and 2 reference genes (Sdha and Pgk1) was performed on the LightCycler® 480 (Roche Applied Science, Indianapolis, IN, USA) to provide an independent evaluation of gene expression. Assays were designed using the Universal Probe Library. All primers and probes have been tested and show linear amplification. The crossing point (Cp) of genes of interest was normalized by the geometric mean of the 2 reference genes.

### Statistical analysis

The difference in expression of each gene by the 10 strains/hybrids was analyzed using one-way ANOVA. The correlations of gene expression levels with previously published data [29] on nicotine SA and food reward were calculated using the mean values for each strain. False discovery rates were used to control for multiple tests.

### Gene co-expression network analysis

WGCNA analysis was conducted using an R package [44]. Modules were constructed using the following parameters: deepSplit = 3, maxBlockSize = 20000, power = 9, networkType = ”signed”, mergeCutHeight = 0.30, minModuleSize = 20, corType  =  “bicor”, pamStage  =  T. “bicor” was used because it is more robust than Pearson correlation [45]. We tested several different mergeCutHeight and minModuleSize values, and similar results were obtained. The modules were correlated with nicotine SA data from the same strains we reported previously. We used the mean nicotine intake obtained during the last 3 days of SA for each strain/hybrid. These 10 values fit a normal distribution, based on the Shapiro-Wilk normality test (p>0.05).

### DAVID gene ontology analysis

The genes in all the modules significantly correlated with nicotine intake were subjected to gene ontology enrichment analysis using DAVID [46]. Over-represented functional categories within each module with an uncorrected EASE score of less than 0.05 were kept as significant as reported previously [47].

### Literature analysis

Chilibot, a literature analysis software that we developed previously [48], identifies genes that are already known to be associated with various smoking behaviors in human genetic studies. The “relationship between two lists” search function was used. The first list contained two keywords: “smoking” and “nicotine”. The official symbols of all the genes in each module were provided as the second list. All modules correlated with nicotine intake; additionally, two modules that did not correlate with nicotine intake were used as controls in Chilibot searches.

## Results

### Measuring gene expression by RNA-seq

Laser capture microdissection was used to obtain tissue from the AcbS of 10 isogenic strains of naïve adolescent male (postnatal day 40–41) rats. Use of this technology ensures precise, reproducible anatomical sampling. On average, 8.0±0.3 ng of total RNA was obtained from each rat. The mean RNA integrity number (theoretical range of 0–10) of the samples was 7.9±0.1 (range: 7.2–8.5), indicating the LCM and extraction process caused minimal RNA degradation. Approximately 30 million 50-bp reads were obtained per sample. Sequences were aligned to the rat reference genome (Baylor 3.4/rn4), using the SHRiMP2 program. On average, 19.3±1.1 million mapped reads were obtained for each sample. The number of mapped reads was not statistically different among the strains (F9,26 = 1.2, P>0.05, see Table S1 for RNA and mapping summaries). Last, gene expression levels, expressed as reads per kilobase per million (RPKM), were normalized across the samples for each brain region and corrected for batch effect (i.e. the date of processing) by using the SampleNetwork script [43]. We retained 12,639 genes, with RPKM values greater than 1 in at least half of the samples, for further analysis.

### Variation in AcbS transcriptome expression: correlation with rat strain, batch and self-administration behavior

We first used one-way ANOVA to identify genes in AcbS that were differentially expressed by rat strain, nicotine SA or food SA. Although 766 genes showed significant differential expression by strain before correcting p values for family-wise error rate, only 16 genes remained at false discovery rate (FDR) of 10% (Table S2). ANOVA also found 1 gene with significant batch effect, defined by the date samples were run, at 10% FDR. Using previously reported nicotine SA and food reward data from these strains [29], we found 801 and 638 differentially expressed genes that correlated with the number of nicotine infusions or food rewards earned, respectively (741 of the 801 were unique to nicotine). None of these genes remained significant at 10% FDR. However, when we restricted our analysis to the small set of 16 genes that were differentially expressed across all rat strains, 2 genes (Cym and Perp) were significantly correlated with nicotine intake (FDR <0.01).

### Detection of co-expression gene networks

**Figure 1:**
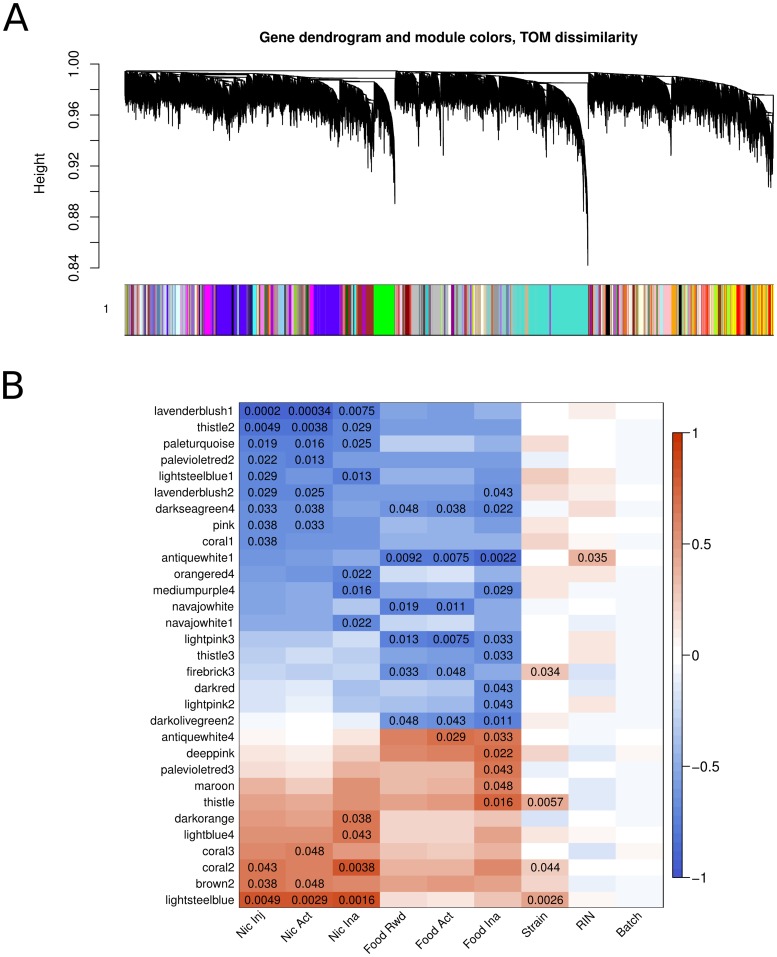
WGCNA detected networks of correlated genes. (A) Weighted Gene Correlation Network Analysis (WGCNA) was applied to 12,639 genes. A total of 127 modules were identified in AcbS. Each module was named after a unique color assigned by the algorithm. Genes within each module were strongly correlated with each other across all the samples. (B) Modules significantly associated with any of the listed traits are plotted. Twelve modules were associated with stable levels of nicotine intake during last 3 d of SA (when nicotine intakes were stable). Of the 12 modules, nine were negatively correlated with nicotine intake, suggesting their expression in the AcbS protect against voluntary nicotine intake in adolescent rodents.

Another biologically meaningful approach to the analysis of transcriptomic data is based on the identification of correlation networks. WGCNA takes a systems biology approach to describe correlation patterns among genes across expression data sets [49]. These correlation patterns yield modules of genes that are highly correlated across samples and may be co-regulated by similar mechanisms. Using WGCNA, we identified 127 gene modules (each named by a unique color; Figure 1A). Together, these modules included 10,733 genes; the remaining 1,906 genes did not belong to any module. Using their eigenvalues (the first principle component, which summarizes the expression pattern of genes within a module), we then correlated each module with various parameters, including the number of nicotine infusions, food reward earned, active lever presses by both nicotine and food SA rats, inactive lever presses by both nicotine and food SA rats, strain, RNA quality (RIN) and batch. All modules that significantly correlated with strain, batch of sequencing run, RNA quality, nicotine or food behavior are shown in Figure 1B. A total of 12 modules were significantly correlated with the amount of stable nicotine intake (i.e., during the last 3 days of the 10 day SA interval). These modules and their genes are reported in Table S3. It is important to note that only 3 of the 12 modules were positively correlated with nicotine intake, while the 9 remaining modules contain genes that are negatively correlated with nicotine intake. Nine of the 12 modules and one additional module (coral3) also were significantly associated with the number of active lever presses elicited to obtain nicotine. Six modules were also correlated with the number of inactive lever presses. This was likely due to an increase in locomotion induced by nicotine during self-administration, which also produced a correlation in the number of presses on the active and inactive levers across strains [29]. Figure 1B also shows that 6 additional modules were correlated with the amount of food reward obtained during food SA. Overall, the majority of these modules correlated with either nicotine or food behavior, demonstrating the behavioral specificity of the modules. Indeed, only 1 module was significantly correlated with both nicotine intake and food reward (i.e., darkseagreen4). It is likely that this module would correlate with general reward. One module was correlated only with inactive lever presses in both nicotine and food sessions (Mediumpurple4), suggesting its potential involvement in locomotion.

We then assessed the biological relevance of these modules by gene ontology enrichment analysis. Among the modules that were correlated with the amount of nicotine intake, Pink contains 244 genes and was enriched in genes related to synaptic plasticity and intracellular signaling. In this module, the number of genes that correlated with nicotine intake was significantly enriched when compared to all genes measured in this study (chi-square test, p<0.05). Table 1 shows the ontology terms pertaining to the Pink module that were related to biological processes and molecular functions. Other modules, such as Coral1, Coral2, Darkseagreen4, Lavenderblush2, Lightsteelblue1, Paleturquoise, and Palevioletred2 were enriched in the following ontology terms: neuron morphogenesis, carbohydrate metabolism, cell adhesion, chromatin binding, organ development, intracellular signaling, and cellular stress response-related genes, respectively. These and other enriched ontologies are shown in Table S4.

**Table 1:** Gene ontology enrichment of the Pink module.

Gene Ontology Term	Fold Enrichment	P value	FDR
GO:0019932∼second-messenger-mediated signaling	5.14	0.000053	0.09
GO:0045761∼regulation of adenylate cyclase activity	8.11	0.000054	0.09
GO:0031279∼regulation of cyclase activity	7.81	0.000069	0.11
GO:0051339∼regulation of lyase activity	7.62	0.000081	0.13
GO:0019933∼cAMP-mediated signaling	7.27	0.000109	0.18
GO:0030817∼regulation of cAMP biosynthetic process	7.27	0.000109	0.18
GO:0030814∼regulation of cAMP metabolic process	7.18	0.000117	0.19
GO:0019935∼cyclic-nucleotide-mediated signaling	6.66	0.000189	0.31
GO:0030808∼regulation of nucleotide biosynthetic process	6.52	0.000216	0.35
GO:0030802∼regulation of cyclic nucleotide biosynthetic process	6.52	0.000216	0.35
GO:0005516∼calmodulin binding	6.30	0.000265	0.37
GO:0030799∼regulation of cyclic nucleotide metabolic process	6.39	0.000245	0.40
GO:0006140∼regulation of nucleotide metabolic process	6.14	0.000312	0.51
GO:0007188∼G-protein signaling, coupled to cAMP nucleotide second messenger	7.38	0.000352	0.57
GO:0007187∼G-protein signaling, coupled to cyclic nucleotide second messenger	6.92	0.000499	0.81
GO:0048666∼neuron development	2.96	0.001434	2.31
GO:0030182∼neuron differentiation	2.59	0.001848	2.97
GO:0009791∼post-embryonic development	5.85	0.003497	5.56
GO:0007190∼activation of adenylate cyclase activity	7.75	0.003792	6.01
GO:0045762∼positive regulation of adenylate cyclase activity	7.46	0.004358	6.88
GO:0030001∼metal ion transport	2.55	0.004870	7.66
GO:0031281∼positive regulation of cyclase activity	7.18	0.004979	7.82
GO:0043506∼regulation of JUN kinase activity	11.29	0.005067	7.96
GO:0042165∼neurotransmitter binding	5.12	0.006173	8.20
GO:0051349∼positive regulation of lyase activity	6.93	0.005656	8.84
GO:0030030∼cell projection organization	2.63	0.005867	9.16

Gene ontology of each module was analyzed using the DAVID function analysis tool. The pink module was enriched with many categories of genes that are involved in synaptic plasticity.

### Validation of RNA-seq data using PCR

**Figure 2:**
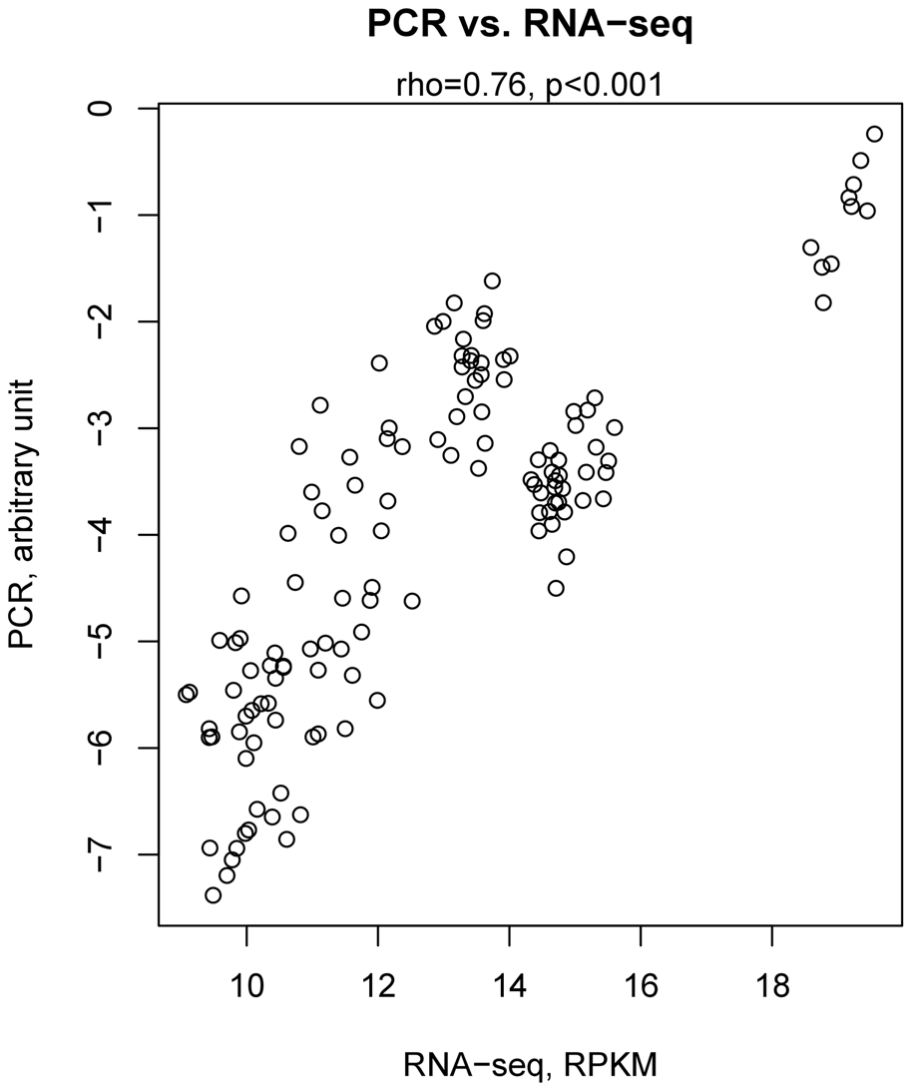
Validation of transcriptome sequencing data using real-time PCR. A total of 13 genes were selected based on the following: strong correlation with the module or nicotine intake; high module membership; strong connectivity within the module. Validation by real-time PCR was conducted using another set of AcbS samples obtained from the same rat strains. The level of gene expression, assayed by transcriptome sequencing vs. PCR across the 10 strains, showed strong correlation (rho  = 0.76 p<0.001).

**Figure 3:**
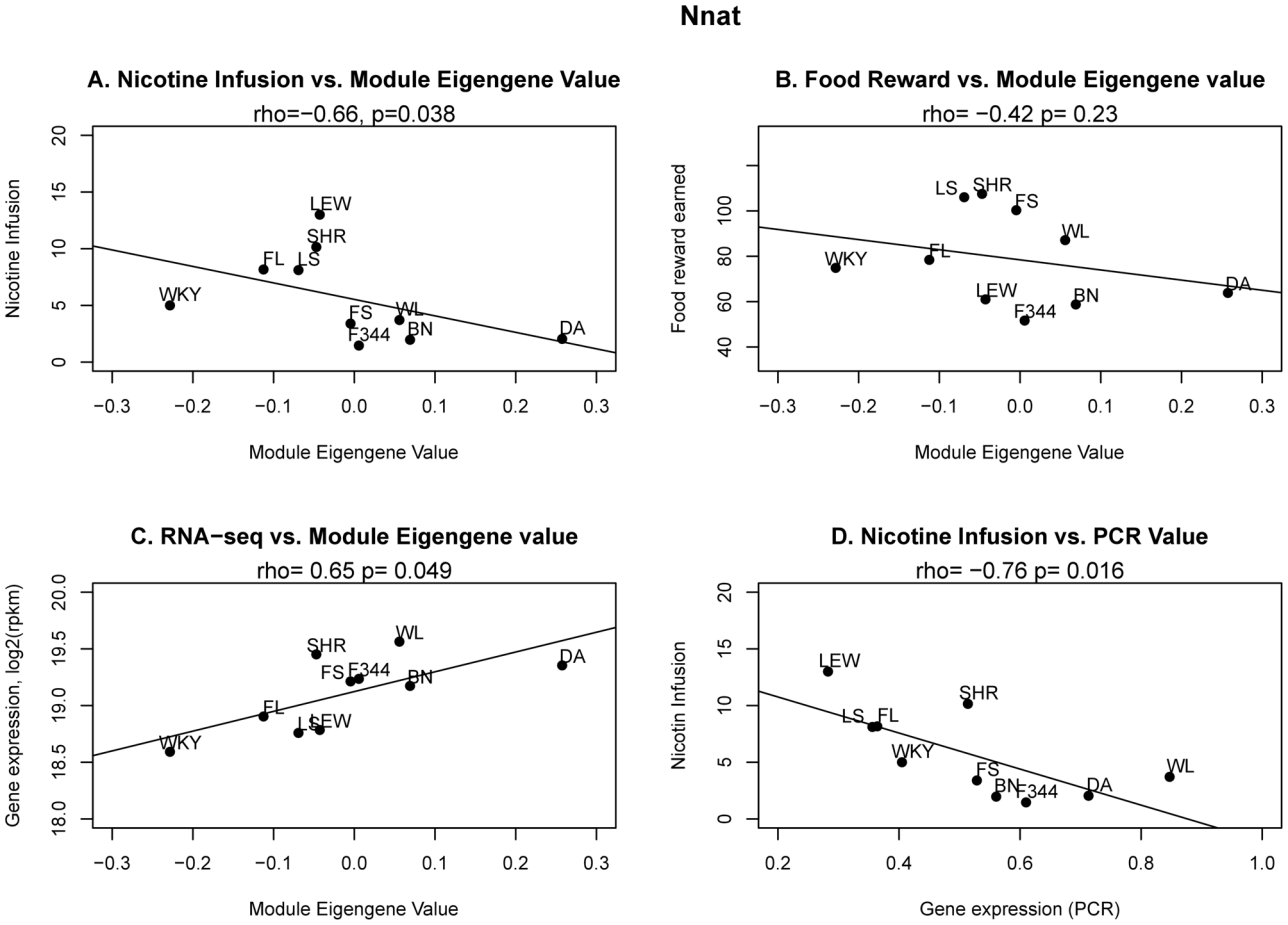
Nnat of the Pink module. The eigenvalue of the Pink module was significantly correlated with nicotine intake (A) but its correlation with food reward earned was not significant (B), suggesting behavioral specificity of the module. Nnat contributed strongly to the expression characteristics of the Pink module (C). The expression pattern of Nnat across the 10 strains measured by PCR showed significant correlation with nicotine intake (D). Strains: BN: Brown Norway, DA: Dark Agouti, F344: Fisher 344, Lew: Lewis, SHR: Spontaneous hypertensive rat, WKY: Wistar-Kyoto. For the F1 hybrids, the two letters representing the initials of the maternal and paternal strains were used.

We validated a select set of genes by PCR quantitation of new AcbS samples obtained from the same rats (3–4 per strain) that had been analyzed by RNA-seq. To prioritize the selection of genes, we considered the following parameters: strong correlation of the module as well as the gene with nicotine intake; high “membership” of the gene in the module (i.e. the extent to which a gene conforms to the characteristic expression pattern of a module) [50]; and strong connectivity of the gene (i.e. the sum of connection weights with all other nodes in the network) [51]. A total of 13 genes that met at least one of the foregoing criteria were selected: Camsap1, Cnot, Gys1, Kat5, Mapk8ip3, Nnat, Pcdha8, Pcdhb9, Ptpn13, Tfg, Unc5a, and Unc5d. A strong correlation was found between PCR and RNAseq data (rho = 0.76, p<0.001; Figure 2). For each of these genes, the correlation of nicotine intake and PCR or RNA-seq data are shown in Table 2. Ten of the 13 genes had shown a significant correlation between expression level (RNA-seq data) and nicotine intake across strains. PCR data validated the RNAseq findings, showing that the basal expression of 6 genes was negatively correlated with nicotine intake. This signifies a role for these genes in protection against the vulnerability to acquire and maintain nicotine SA behavior. The expression of a representative gene, neuronatin (Nnat), a member of the Pink module, was correlated with nicotine intake (Figure 3A), but not earned food reward (Figure 3B). Nnat was a strong member of the module, as shown by the fact that its expression pattern across strains had a high correlation with its module eigengene values (Figure 3C). PCR data confirmed that Nnat expression in AcbS was negatively correlated with nicotine intake (Figure 3D).

**Table 2:** Correlation of nicotine intake with gene expression measured by PCR in AcbS.

Gene	RNA-seq	Column1	PCR	Column2
	Rho	p	Rho	p
**Camsap1**	0.79	0.007	−0.50	0.143
**Cnot4**	0.77	0.014	−0.26	0.470
**Gys1**	−0.83	0.006	−0.71	0.028
**Kat5**	−0.68	0.035	−0.12	0.759
**Mapk8ip3**	−0.73	0.021	−0.35	0.331
**Nnat**	−0.30	0.407	−0.76	0.016
**Pcdha8**	−0.62	0.060	−0.35	0.331
**Pcdhb9**	−0.83	0.006	−0.85	0.004
**Ptpn12**	0.72	0.024	−0.58	0.088
**Ptpn13**	−0.55	0.104	−0.66	0.044
**Tfg**	−0.83	0.006	−0.72	0.024
**Unc5a**	−0.70	0.031	−0.78	0.012
**Unc5d**	−0.71	0.021	−0.45	0.191

A total of 13 genes were selected for validation. The levels of gene expression obtained by RNAseq vs. real-time PCR were correlated with the amount of nicotine intake from the same strains (data reported previously [29]).

### Identifying genes putatively related to cigarette smoking in the literature

We used Chilibot, a literature analysis software that analyzes PubMed abstracts [48], to identify genes that have been potentially associated with smoking in human genetic studies. We identified 15 genes in the Pink module that human studies have potentially associated with various smoking-related behaviors, including smoking initiation, nicotine dependence and smoking cessation, etc. (Table 3). We also examined 2 other modules (i.e., Purple and Greenyellow) that were similar in size to the Pink (>200 genes each) module, but were not correlated with nicotine intake. Chilibot found only 1 gene in the Purple module (i.e., RAB4B [52]) that has been associated with smoking.

**Table 3:** Genes of modules correlated with nicotine intake that were implicated in smoking behavior.

Gene	membership	Connectivity	Smoking phenotype	References
Nnat	0.85	0.77	Nicotine dependence	[53]
Grin3a	0.75	0.19	Nicotine dependence	[54]
Pde1a	0.72	0.53	Smoking cessation	[4]
Sema5a	0.70	0.11	Smoking cessation	[55]
Kcnip4	0.70	0.21	Smoking cessation	[55]
Nrxn1	0.70	0.37	Nicotine dependence	[56]–[59]
Galr1	0.68	0.13	Smoking cessation	[60]
Dscaml1	0.63	0.15	Smoking cessation	[55]
Map3k4	0.63	0.08	Nicotine dependence	[61]
Grm8	0.63	0.15	Smoking initiation	[62]
Cdkn1a	0.61	0.06	Smoking quantity	[63], [64]
Ppp1r1b	0.58	0.01	Smoking quantity	[65]
Chrm5	0.55	0.16	Smoking quantity	[66]
St6galnac3	0.51	0.06	Smoking cessation	[55]
Fgfr1	0.27	0.01	Smoking initiation	[67], [68]

A literature mining software, Chilibot [48], was used to search for genes reported to be involved in smoking behavior. Manually curated results confirmed that 15 genes of the Pink module were potentially associated with smoking behaviors. Searching 2 additional modules with similar numbers of genes found only 1 gene potentially associated with smoking behavior.

## Discussion

We sequenced the transcriptome of AcbS samples obtained by LCM from 10 isogenic strains of naïve adolescent rats and compared the RNA transcript levels with nicotine SA behavior, reported previously [29], using WGCNA. Twelve gene modules were significantly associated with the amount of self-administered nicotine, nine of which were negatively correlated. Six modules were correlated with food reward. Only one module was related to both nicotine and food, demonstrating the behavioral specificity of the modules. The associations of selected genes with nicotine intake were confirmed by PCR. Chilibot, a literature mining tool [48], identified 15 genes within the Pink module that are potentially associated with cigarette smoking in human studies, thereby providing strong support for the primary analytical approach (i.e., WGCNA) taken in this study. This is the first report demonstrating that motivated intake of i.v. nicotine by adolescent rodents is associated with the basal expression levels of specific genes in the mesolimbic circuitry [30], [31] underlying motivated drug-taking behaviors.

The vulnerability to acquire and maintain nicotine SA can be conceived of as a balance between genes, with differential basal expression, conferring either susceptibility or resistance in each strain. Therefore, strong protection against this vulnerability reflects the combined effects of reduced expression of susceptibility genes and increased expression of resistance genes. In this study, we identified 9 gene expression modules that provide protection against nicotine SA due to reduced expression of certain genes across the 10 isogenic rat strains. These modules were negatively associated with the levels of nicotine SA behavior that we reported previously [29]. A systematic literature search of human studies found 15 genes that are potentially involved in smoking behavior. However, in many cases, the published reports did not provide insight into the specific effects of these genes on smoking behavior nor was their statistical significance for specific smoking phenotypes explicitly confirmed at the population level. In contrast, the intrinsic design of this study, based on the quantitative analysis of individual gene expression levels across 10 isogenic strains and their correlations with nicotine intake, permits us to characterize these genes as protection genes with respect to their function within the adolescent AcbS.

Insight into the unique biological function of the genes grouped within a module was obtained by detecting the enrichment of ontological terms [69]. Eight of the 12 modules (e.g., Pink module, Table 1) that correlated with nicotine intake were also significantly enriched in genes belonging to specific ontological categories that are subsets of more general terms such as synaptic plasticity and intracellular signaling pathways. Additionally, modules were enriched in genes related to the following ontological terms: neuron morphogenesis, carbohydrate metabolism, cell adhesion, chromatin binding, and cell proliferation (Table S4). Although the function of most genes is still poorly understood, thereby limiting the specificity of their annotation, the enrichment in ontological terms enabled both the validation and annotation of modules that would otherwise only be defined mathematically.

We focused on Pink, the largest module negatively correlated with nicotine intake (244 genes), because of features that are directly related to nicotine and human smoking. Table 1 shows that Pink was enriched in genes involved in intracellular signaling and synaptic plasticity, such as cAMP-mediated signaling (e.g., Calcr, Galr1, Grm8, Gnai1), calmodulin binding (e.g., Camk1g, Atp2b4, Trpv1) and neuronal differentiation (e.g., Ctf1, Fgfr1, Grin3a, Neurod4, Nnat), etc. Nnat, a neuronal differentiation gene in Pink module, was significantly correlated with its eigengene values (Figure 3C), demonstrating that it is a major determinant of this module. PCR measurements of AcbS samples confirmed that Nnat expression was negatively correlated with nicotine intake (Figure 3D). Developmentally regulated, Nnat is highly expressed during neurogenesis [70], and it may influence site-specific synaptic events such as long-term potentiation or depression by modulating local dendritic calcium levels [71]. Perhaps most importantly, a recent genetic linkage study, using DSM-IV criteria, identified chromosome 20q11.23 as a locus harboring genetic variations linked to nicotine dependence [53]. NNAT is one of the genes located in this region. Although this study [53] did not specifically assess polymorphisms in the NNAT gene, our data show that Nnat is potentially involved in driving the linkage of this chromosomal region with nicotine dependence (i.e., at least in women).

In addition to Nnat, 14 other Pink module genes (Table 3), potentially associated with human smoking, were identified by Chilibot, a literature-mining program developed in our laboratory [48]. N-methyl-D-aspartate receptor 3A (Grin3a), like Nnat, is involved in neuronal differentiation [72]. In a candidate gene study, both individual SNP and haplotype association tests of African-Americans and European-Americans revealed significant associations of Grin3A with nicotine dependence [54]. Also found in Pink module, the regulatory subunit 1B of protein phosphatase 1 [Ppp1r1b (a.k.a. DARPP-32)], a major target of striatal dopamine, modulates protein kinase A and protein phosphatase-1, both of which are critical mediators of electrophysiological and transcriptional responses to multiple drugs of abuse [73]. Low dose nicotine (1 µM) reduced Thr34 phosphorylation of Ppp1r1b, whereas high dose (100 µM) increased its phosphorylation at multiple sites [74], [75]. Moreover, a significant association of Ppp1r1b haplotype with smoking intensity was found in European-Americans [65]. A third example, Neurexin 1 (Nrxn1), is a cell adhesion molecule involved in synaptogenesis [76] and synaptic plasticity [77]. A genome-wide association study first posited a role for NRXN1 in nicotine dependence [56]. Independent studies have confirmed this association in European- and African-American [57] as well as Japanese [58] smokers. A meta-analysis of genome-wide linkage scans also confirmed the association of NRXN1 with nicotine dependence [59]. Other genes in the Pink module are known to be involved in the neurobiological effects of nicotine. For example, the serotonin receptor 1 A (Htr1A) is modulated by chronic nicotine exposure [78], [79], and visinin-like 1, a neuronal calcium-sensing protein involved in nicotine-induced calcium signaling, induced the up-regulation of alpha4beta2 nicotinic acetylcholine receptors [80], [81]. In summary, the Pink module, which is inversely correlated with nicotine intake in our study, contains 15 genes that are potentially associated with human smoking and the neuronal effects of nicotine.

The identification of these 15 genes within a single module, the Pink module, is important for several reasons. First, by showing that a large group of genes previously associated with human smoking are linked together and directly correlated with nicotine intake across 10 isogenic strains of rat, we validate both the application of WGCNA to a transcriptomal data set and the overall approach of using brain region-specific tissue derived from isogenic strains that differ in their nicotine SA behavior. These genes are linked within a single module by their significant co-variance, although the underlying biological drive has yet to be defined. Second, the target brain region (i.e., AcbS) for the activity of these 15 genes, which cannot be inferred from human genetic studies, is defined by this study. Third, the results of this study strongly suggest that the basal expression of these 15 genes in AcbS is critical to protecting against the initiation and maintenance of smoking in adolescents. It is also likely that the expression of some of these genes may be modulated by nicotine SA. Fourth, other genes in the Pink module, as well as other nicotine-correlated modules, are also likely to contribute to the regulation of smoking behavior in humans. Last, using the same methods, we conducted a literature search on 2 modules that were not correlated with nicotine intake but were similar in size to the Pink module. We found only 1 gene previously implicated in smoking, thus further demonstrating the significance of the findings derived from the Pink module.

We further confirmed our results by selecting individual genes from multiple modules and validating their expression levels by real-time PCR, using a separate set of brain sections obtained from the same rats used in the RNA-seq. We found strong correlations between RNA-seq and PCR data (Figure 2). The correlations between gene expression levels and nicotine intake were also validated for most genes (Table 2). Many of these confirmed genes contribute to the enrichment of the gene ontology categories in their respective modules. However, the correlations between nicotine intake and the expression levels of several genes, measured using RNA-seq, were not replicated by the PCR data. This most likely reflects the fact that each rat strain (i.e., all the individual members of the strain) was treated as one sample in our analysis, despite the fact that the value for each strain was obtained from multiple animals. Although 10 is a relatively large and practical number of strains for studying transcriptomal changes, it is likely that this number is still not sufficient to produce truly robust results in a correlation analysis.

Similarly, when genes were tested individually for their correlation with nicotine SA, none of the nominally significant genes passed a genome wide correction for p values. If we assume that the large difference in nicotine intake observed among the 10 isogenic strains (i.e. 8.9 fold) was partially determined by differential gene expression within AcbS, which is well supported by numerous studies [28], [30]–[32], [82], this lack of statistical significance is likely due primarily to the relatively limited number of strains that we were able to analyze at the practical scale of this work. Because the correlation between the expression level of each gene across strains and behavior was analyzed using the mean values from each strain, the relatively small sample size (i.e., n = 10) would impose limits on the statistical power to detect transcriptomic variation vs. behavior. This is especially so because we minimized between-animal behavioral variation by studying isogenic animals bred in our laboratory (i.e., to avoid stress caused by long distance shipping), used precise anatomical sampling by laser capture microdissection, and relied on a validated [38], highly precise, state-of-the-art sequencing method to measure gene expression levels.

In summary, using anatomically precise tissue obtained from AcbS and a state-of-the-art gene expression profiling method, we analyzed the correlation of genome-wide gene expression profiles with voluntary intravenous nicotine intake by 10 isogenic strains of adolescent rat. We identified 12 gene modules that were correlated with the amount of nicotine intake, 9 of which protected against the acquisition of nicotine SA behavior. Among them, one module (i.e., Pink) was enriched with 15 genes that are potentially associated with cigarette smoking in humans. This overlap between rodent and human data not only validated our experimental approach, but it provided potential mechanisms, reflecting the functions of and interactions between candidate genes within a module. Hence, individual genes within a module appear to regulate specific molecular processes within AcbS neurons and the interaction of these processes controls the functional activity of neurons and the AcbS network.

## Supporting Information

Table S1 Sample RNA quality and mapping statistics.
(XLS)

Table S2 Genes differ by strain.
(XLS)

Table S3 Modules that correlated with nicotine intake and their genes.
(XLS)

Table S4 Enrichment of Gene Ontology Categories for Nicotine Correlated Modules.
(XLS)
